# Needle Types and Diagnostic Accuracy in EUS-Guided Liver Biopsy: A Systematic Review and Network Meta-Analysis

**DOI:** 10.3390/diagnostics16121857

**Published:** 2026-06-16

**Authors:** Antonio Facciorusso, Mohammed Albeshir, Mohammed S. AlQahtani, Stefano Francesco Crinò, Giuseppe Dell’Anna, Gianfranco Donatelli, Marcello Maida, Mattia Brigida, Elisa Stasi, Armando Dell’Anna, Emad S. Aljahdli, Eyad Gadour

**Affiliations:** 1 Gastroenterology Unit, Università del Salento, 73100 Lecce, Italy; mattiabrigida@hotmail.it (M.B.); elisastasi2@gmail.com (E.S.); armando.dellanna@alice.it (A.D.); 2 Division of Gastroenterology, Department of Medicine, King Fahad Specialist Hospital, Dammam 32253, Saudi Arabia; albeshirm@outlook.com; 3 Department of Surgery, Imam Abdulrahman Ibn Faisal University, Dammam 31441, Saudi Arabia; alqahtanim222@gmail.com; 4 Multi-Organ Transplant Centre of Excellence, Liver Transplantation Unit, King Fahad Specialist Hospital, Dammam 32253, Saudi Arabia; eyadgadour@doctors.org.uk; 5 Department of Medicine, Università di Verona, 37134 Verona, Italy; stefanocrino@hotmail.com; 6 Gastroenterology and Endoscopy Unit, IRCCS Policlinico San Donato, 20097 Milan, Italy; dellanna.giuseppe@hsr.it; 7 Faculty of Medicine, Università Vita-Salute San Raffaele, 20132 Milan, Italy; 8 Interventional Endoscopy Unit, Private Hospital Peupliers, Ramsay Santé, 75013 Paris, France; donatelligianfranco@gmail.com; 9 Department of Clinical Medicine and Surgery, University of Naples “Federico II”, 80138 Naples, Italy; 10 Department of Medicine and Surgery, University of Enna ‘Kore’, 94100 Enna, Italy; marcello.maida@unikore.it; 11 Gastroenterology Division, Faculty of Medicine, King Abdulaziz University, Jeddah 21589, Saudi Arabia; ealjahdli@gmail.com; 12 Department of Medicine, Faculty of Medicine, Zamzam University College, Khartoum 11113, Sudan

**Keywords:** GRADE, EUS, adequacy, diagnosis, hepatic

## Abstract

**Background:** The optimal needle for endoscopic ultrasound (EUS)-guided liver biopsy (LB) remains uncertain. **Methods:** We conducted a network meta-analysis that integrated both direct and indirect comparisons among various needles. Our analysis included five randomized controlled trials (RCTs) involving 251 patients, which compared 19G fine-needle aspiration (FNA), 19G Franseen fine-needle biopsy (FNB), 19G Fork-tip FNB, 22G Franseen FNB, and 22G Fork-tip FNB. The primary outcomes focused on the total specimen length and the number of complete portal tracts (CPTs). Results were presented as mean difference (MD) or risk ratio (RR) with 95% confidence intervals (CIs). **Results:** The 19G Fork-tip and 19G Franseen needles were found to be significantly superior to the others in terms of total specimen length and CPT count. No differences were observed between the 19G FNA and the 22G Franseen or Fork-tip needles. SUCRA ranking indicated that the 19G Franseen and 19G Fork-tip FNB were the top-performing needles, with SUCRA scores of 0.91 and 0.83 for specimen length, and 0.85 and 0.82 for CPTs. No severe adverse events were reported. The quality of evidence was generally considered low due to bias risk and imprecision. **Conclusions:** The 19G Franseen and Fork-tip FNB showed favorable results but further RCTs are needed to confirm these results.

## 1. Introduction

While the widespread use of non-invasive tests has streamlined the diagnostic approach to several liver diseases, histological assessment through liver biopsy (LB) is still frequently needed [1].

The main goal of a liver biopsy is to obtain an adequate specimen for diagnosis. The American Association for the Study of Liver Diseases (AASLD) defines an optimal specimen as at least 2–3 cm long after formalin fixation and containing ≥11 complete portal tracts (CPTs) [2]. On the other hand, a more recent guideline by the British Joint Societies recommends achieving samples >20 mm in length but irrespective of the number of CPTs [1].

Percutaneous liver biopsy, usually under ultrasound guidance, has represented for decades the gold standard for liver tissue sampling. Trans-jugular liver biopsy could represent a valuable alternative option in patients with end-stage liver disease, coagulation impairment or ascites, since its transvenous approach and lack of capsule perforation could decrease the risk of hemorrhagic complications [3].

Endoscopic ultrasound-guided (EUS) tissue acquisition through fine-needle aspiration (FNA) or fine-needle biopsy (FNB) already plays a pivotal role in the diagnostic algorithms of pancreatic and abdominal solid lesions [4,5,6] and EUS-LB has shown a favorable diagnostic yield with a reported histologic adequacy rate of 93.9% and a low complication rate of 2.3% [7]. These promising results were further improved with newer end-cutting FNB needles, such as the Franseen needle (Acquire^®^ [Boston Scientific, Marlborough, MA, USA]) and the Fork-tip needle (SharkCore^®^ [Medtronic, Dublin, Ireland]) and EUS-LB has been recently proposed as an alternative to percutaneous or trans-jugular LB with similar results, lower postoperative pain scores and the possibility to also reach deep or difficult locations for tissue sampling [8,9].

Although EUS-LB is currently a well-established and widely used technique, it is still unclear which needle should be chosen. A previous meta-analysis found that the diagnostic yield of 19G FNA needles was higher than that of core needles, mainly reverse bevel FNB [7]. On the other hand, a more recent meta-analysis showed a higher number of CPTs using FNB needles than FNA [10]. Furthermore, among end-cutting FNB needles, the Franseen needle was found to performed better than the Fork-tip device in another meta-analysis [11].

Therefore, given the discordant results in the literature, we decided to perform a systematic review and network meta-analysis of available randomized controlled trials (RCTs) comparing different needles for EUS-LB.

In contrast to pairwise meta-analyses, network meta-analysis can provide also indirect evidence about the comparative effectiveness of multiple interventions and synthesize available data across a network of RCTs.

We also used Grading of Recommendations Assessment, Development and Evaluation (GRADE) criteria [12] for network meta-analysis to appraise the quality of evidence.

## 2. Methods

This systematic review is reported according to the Preferred Reporting Items for Systematic Reviews and Meta-Analyses (PRISMA) extension statement for network meta-analyses statements [13].

### 2.1. Selection Criteria

Studies included in this meta-analysis were RCTs published in the English language fulfilling the following inclusion criteria: (a) Patients: adults (age > 18 years) undergoing EUS-LB; (b) Interventions: 19G FNA, 19G Franseen FNB, 19G Fork-tip FNB, 22G Franseen FNB, 22G Fork-tip FNB; (c) Comparators: another of the above reported needles; and (d) Outcomes: total specimen length, number of CPTs, diagnostic adequacy, adverse events (AEs). Non-comparative observational and retrospective studies, trials comparing different sampling strategies or number of actuations/passes within the same needles, or RCTs comparing EUS-LB to percutaneous LB were excluded.

### 2.2. Search Strategy

The search strategy used various databases with variant controlled vocabularies, expanded terminology, varying algorithms, and keyword capabilities, for RCTs published from inception through May 2025. Complementary manual search was performed on additional databases and by checking the references of all the main review articles on this topic, in order to identify possible additional studies. The detailed literature search strategy is reported in the Supplementary Materials (Table S1). The review was not registered in an international registry.

Data were abstracted onto a standardized form by two authors independently. The risk of bias of individual studies was assessed using the Cochrane Risk of Bias assessment tool [14] by two authors. In the case of disagreements, the decision was based on a third author’s opinion.

### 2.3. Outcomes

Primary outcomes were the total specimen length, measured in mm, and the mean number of CPTs. Secondary outcome was the diagnostic adequacy of the sample, defined mainly as at least 2 cm of specimen length with ≥11 CPTs [2]. AEs were inconsistently reported and were analyzed only descriptively [15].

### 2.4. Statistical Analysis

The network meta-analysis was performed using a multivariate random-effects meta-regression with a frequentist approach based on a random-effects consistency model and the results were expressed as mean difference (MD) or risk ratio (RR) along with 95% confidence intervals (CIs). A comparison of direct and indirect treatment estimates was performed (when available) to assess the consistency of network meta-analyses by using a node-splitting technique. We assessed the statistical heterogeneity using I^2^ statistic, with values over 50% indicating substantial heterogeneity. Funnel plots could not be reported given the limited number of studies [16].

Methods ranking was based on the surface under the cumulative ranking curve (SUCRA) score analysis, measured on a scale from 0 (worst) to 1 (best) [17].

Network meta-analysis was conducted with R package *netmeta* 3.5 (Foundation for Statistical Computing, Vienna, Austria).

### 2.5. Quality of Evidence

The quality of evidence derived from the pairwise and network meta-analyses was judged using the GRADE framework [12]. In this approach, the evidence from RCTs starts at high quality and can be rated down based on risk of bias, indirectness, imprecision, inconsistency (or heterogeneity) and/or publication bias, to levels of moderate, low and very low quality (Table 1 and Table S2).

## 3. Results

**Figure 1:**
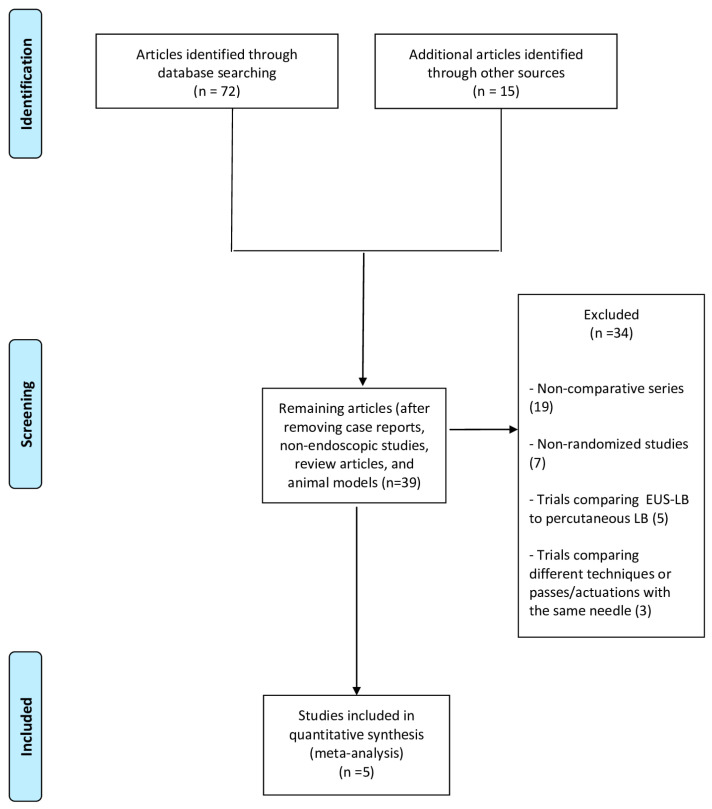
Flow chart of the included trials.

**Figure 2:**
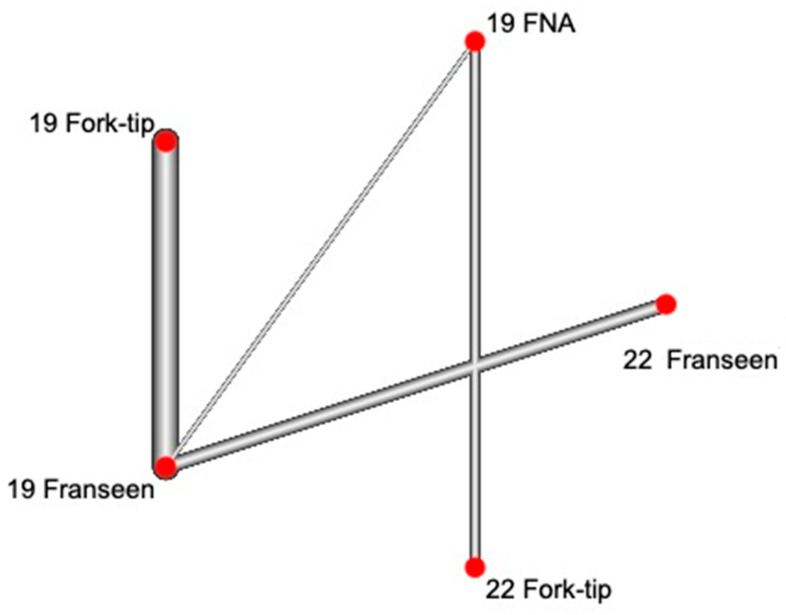
Network of the included trials. Network of included studies with the available direct comparisons between needles for EUS-guided liver biopsy. The size of the nodes and the thickness of the edges are weighted according to the number of studies evaluating each treatment and direct comparison, respectively. FNA: fine-needle aspiration.

Out of 87 articles initially identified using the search strategy, we finally included five RCTs in the network meta-analysis, comparing five different needles (19G FNA, 19G Franseen FNB, 19G Fork-tip FNB, 22G Franseen FNB, 22G Fork-tip FNB) for EUS-LB [18,19,20,21,22]. Figure 1 details the study selection flowchart, and Figure 2 shows the available direct comparisons and network of trials. All the comparisons were informed by a single RCT, except the comparison between 19G Franseen and 19G Fork-tip needles which was based on two RCTs [20,21]. 19G Franseen was the main common comparator in the indirect comparisons.

### 3.1. Characteristics of Included Studies

Table 2 summarizes the baseline characteristics of the RCTs included in the network meta-analysis. Overall, the included five trials enrolled 251 patients. Two RCTs compared 19G Franseen vs. 19G Fork-tip [20,21], one RCT compared 19G FNA vs. 19G Franseen [18], one RCT compared 19G FNA vs. 22G Fork-tip [19], and one RCT compared 19G Franseen vs. 22G Franseen [22].

Three studies [18,20,22] complied strictly to the standard definition of diagnostic adequacy (at least 2 cm of specimen length with ≥11 CPTs), one RCT [19] defined the adequacy as ≥5 CPTs, and one RCT [21] did not report a minimum number of CPTs to define the adequacy.

The baseline patient characteristics were homogeneously distributed between the active and comparator groups and across different trials (Table 2). All the RCTs included only EUS-LB for parenchymal liver disease.

The recruitment period ranged from 2016 to 2022 and all the studies were conducted in the USA. In three RCTs both liver lobes were sampled [18,19,22], and the number of needle passes and actuations ranged from 1 to 4 and 1 to 7, respectively. The wet suction (mainly with heparin) was used in all the included RCTs.

As described in Figure S1A,B, all studies were at high risk of performance bias. Moreover, one RCT [21] was considered also at high risk of selection bias due to the unconcealed sequence generation and allocation of patients.

### 3.2. Total Specimen Length

**Figure 3:**
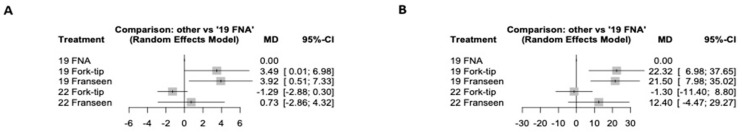
Forest plots reporting estimates derived from network meta-analyses assessing (**A**) total specimen length, and (**B**) complete portal tracts. 19 fine-needle aspiration was used as reference. Results were expressed in terms of mean difference and 95% confidence intervals.

As reported in Table 1, total specimen length for both 19G Fork-tip and 19G Franseen FNB were significantly superior as compared to the other needles (MD 2.76, 1.43 to 4.10 and 3.19, 2.07 to 4.31, respectively vs. 22G Franseen FNB; MD 4.78, 0.95 to 8.62 and 5.21, 1.45 to 8.97, respectively vs. 22G Fork-tip; MD 3.92, 0.51 to 7.33 and 3.49, 0.01 to 6.98, respectively vs. 19G FNA). No difference between 19G FNA and 22G Franseen or Fork-tip needles was detected (Table 1 and Figure 3A). Likewise, 22G Franseen and Fork-tip FNB needles did not show any difference (MD −2.02, −5.95 to 1.91). I^2^ was 0%, suggesting the absence of heterogeneity.

As a consequence, as reported in Table 3, SUCRA ranking suggested 19G Franseen and 19G Fork-tip FNB as the best performing needles (SUCRA 0.91 and 0.83, respectively), with 19G FNA and 22G Fork-tip FNB showing the poorest yield (SUCRA 0.24 and 0.09, respectively).

### 3.3. Number of CPTs

On network meta-analyses, 19G Franseen and 19G Fork-tip FNB significantly outperformed 22G Fork-tip FNB (MD 23.62, 5.25 to 41.98 and 22.80, 5.92 to 39.68, respectively) and 19G FNA (MD 21.50, 7.98 to 35.02 and 22.32, 6.98 to 37.65, respectively; Table 1 and Figure 3B). Notably, no statistical difference was observed between 19G Franseen and 19G Fork-tip FNB vs. 22G Franseen FNB (MD 9.92, −2.49 to 22.32 and 9.10, −0.98 to 19.18, respectively). No other differences were detected (Table 1). I^2^ was 0%, suggesting the absence of heterogeneity.

As a consequence, again 19G Fork-tip and 19G Franseen FNB were the best needles in terms of CPTs (SUCRA 0.85 and 0.82, respectively), followed by 22G Franseen (SUCRA 0.53), 19G FNA (SUCRA 0.17) and finally 22G Fork-tip (SUCRA 0.10) (Table 3).

### 3.4. Diagnostic Adequacy and Adverse Events

As reported in Tables S3 and S4, 19G Fork-tip and 19G Franseen FNB were significantly superior to 22G Franseen in terms of diagnostic adequacy (RR 6.61, 1.08 to 40.53 and RR 8.50, 1.76 to 41.09, respectively). I^2^ was 0%, suggesting the absence of heterogeneity. No other differences were detected. As a consequence, 19G Franseen and 19G Fork-tip FNB were the best performing needles (SUCRA 0.81 and 0.63, respectively) whereas 22G Franseen FNB showed the poorest adequacy rate (SUCRA 0.02).

Table 2 descriptively reports the AEs registered in the included studies. Only one case of gastric hematoma was observed in the study by Aggarwal et al. [21] whereas a varying range of episodes of mild abdominal pain was reported in the other RCTs.

### 3.5. Network Coherence and Quality of Evidence

There were no significant differences between direct and indirect estimates in closed loops that allowed assessment of network coherence (*p*-values for differences between groups where both direct and indirect estimates were available ranging from 0.21 to 0.45 for total specimen length and from 0.15 to 0.72 for number of CPTs).

The quality of evidence was mainly judged as low due to the risk of bias in the literature and imprecision (broad 95% CIs crossing the unity or low number of events not reaching the required information size). In the analysis of diagnostic adequacy, the quality of evidence was further downgraded to very low due to the additional risk of indirectness related to the different definitions of adequate sample across the included RCTs.

## 4. Discussion

Other non-invasive modalities have not yet superseded completely liver biopsy in the diagnostic algorithm of liver disease, particularly in conditions such as autoimmune hepatitis or some focal lesions when proper histology and immunohistochemistry are required. To overcome the main limitations of percutaneous LB, such as the risk of potentially serious complications, inter-observer variations, and sampling errors, trans-jugular LB has been tested in high-risk patients, such as those with coagulopathy, in antithrombotic therapy, or high-volume ascites; however, the complexity of the procedure and the potential risks for complications limit its applicability.

The EUS-LB approach enables achieving samples from both hepatic lobes even in deep locations and EUS guidance can improve the safety profile of the procedure and minimize the impact of ascites and body habitus on the diagnostic yield.

However, which is the best needle for EUS-LB is still unclear. Results of previous pairwise meta-analyses were discordant [7,10,11], and several RCTs have been published in the latest years particularly testing newer end-cutting FNB needles.

Through a network meta-analysis, to our knowledge the first ever published in the field, and using GRADE criteria to appraise the quality of evidence, we made several key observations. First, 19G end-cutting FNB, either Fork-tip and Franseen, enabled us to achieve significantly longer samples with a higher number of CPTs as compared to the other needles. On the other hand, no difference between 19G FNA and 22G end-cutting FNB needles was observed. As expected, 22G FNB needles performed equally (MD −2.02, −5.95 to 1.91).

Therefore, 19G end-cutting FNB appeared to determine more favorable results as suggested in the SUCRA ranking, with 19G FNA and 22G Fork-tip FNB showing the poorest yield.

Our results suggest that larger caliber needles are needed to achieve longer samples with more CPTs that are fundamental for the proper diagnosis of several liver diseases. However, the larger size needles could raise some concerns about the maneuverability of the needle and this should be considered particularly by less experienced operators. While the 22G end-cutting FNB needles were initially tested with promising results in some retrospective studies [23,24,25], our network meta-analysis and two RCTs clearly suggest the use of larger size FNB needles to obtain high-quality samples [19,22]. Unfortunately, comparative data on another large size end-cutting FNB needle, namely the 20G forward bevel FNB, are lacking and it needs to be tested in future RCTs following the promising results of a preliminary study [25].

As the second finding of note, 19G FNB resulted significantly superior to 22G Franseen but not to 19G FNA in terms of diagnostic adequacy, although these two needles still ranked as the best performing devices as for this specific parameter. The definition of diagnostic adequacy is inconsistent across the included RCTs and it relies on different definitions particularly concerning different cut-offs for the number of CPTs. Therefore, while 19G FNA needles led to similar rates of adequate samples as compared to 19G end-cutting FNB, the quality of these samples was significantly superior, with the latter thus paving the way to their broader use in the clinical practice. Moreover, the analysis of diagnostic adequacy should be interpreted with caution and the findings should be considered as exploratory due to the very low quality of evidence of the results, mainly related to the inconsistent definition of adequacy used in the included RCTs. Interestingly, all the RCTs used the wet-suction technique that already proved to perform properly in other abdominal masses [4]. It would be of interest to test other sampling techniques although preliminary results speak in favor of the heparinized wet-suction approach for EUS-LB [26].

The third remarkable finding in our study was the favorable safety profile of all the needles tested with only one case of gastric hematoma observed in the study by Aggarwal et al. [21] and some episodes of mild abdominal pain reported in the other RCTs. While the use of larger size needles could raise some concerns on the maneuverability of the needle and the safety of the procedure, no case of technical failure nor of severe AE was reported. However, it should be noted that the included RCTs were underpowered to detect these uncommon events and reliable comparative safety assessment requires larger studies. Therefore, AE reporting in our study was descriptive and inconsistent across studies.

Finally, the low quality of the evidence due to the risk of performance bias in the literature and the imprecision of the results calls for further RCTs to confirm these results and to strengthen the recommendation in favor of 19G FNB needles to inform the forthcoming clinical guidelines.

There are certain limitations, related to both the network analysis, as well as individual studies, which merit further discussion. First, the paucity of direct head-to-head trials for certain comparisons resulted in low- to very low-quality evidence for comparative efficacy of the different needles. It should be noted that multiple indirect comparisons in the absence of a good number of head-to-head RCTs can lead to weaker conclusions that can sometimes be erroneous, specifically concerning the interpretation of the SUCRA ranking. Particularly, SUCRA ranking should be interpreted with caution because the limited number of RCTs could bias the results towards the most represented intervention in the network. Second, network meta-analyses may be subject to misinterpretation due to conceptual heterogeneity, related to considerable differences in participants, interventions, co-interventions/background treatment and outcome assessment, which may limit comparability of trials. For example, the included RCTs used different approaches to sampling, whether bilobar or only right lobe sampling. Which is the best approach is still unclear and was beyond the scope of the current meta-analysis; moreover, the impact of these potential effect modifiers is uncertain. However, their potential impact on the indirectness of our results was considered in the analysis. Moreover, as already mentioned above, the definition of diagnostic adequacy was heterogeneous in the different RCTs and the limited number of studies prevented an adequate sensitivity analysis based on this aspect; however, the primary outcomes were specifically focused on the defining features of adequacy, namely specimen length and number of CPTs, so we think this heterogeneity did not affect our results.

Regarding limitations of individual studies, we acknowledge that most studies had a small sample size and did not explore all the potential confounders, for example, the aforementioned approach to sampling (whether one-lobe or bilobar) nor did they test the impact of ancillary techniques, rapid on-site evaluation (ROSE) or macroscopic on-site evaluation (MOSE) on EUS-LB. Moreover, all the RCTs were conducted in the USA and this could affect the reproducibility of the results.

However, in spite of these limitations, to the best of our knowledge this manuscript represents the first network meta-analysis to compare different needles for EUS-LB. While robust GRADE methodology demonstrated mainly a low level of evidence, we observed that 19G end-cutting FNB needles appear to provide the highest histologic yield based on currently available evidence. Further RCTs are needed to confirm these results.

## 5. Conclusions

This network meta-analysis demonstrates that 19G end-cutting fine-needle biopsy (FNB) needles, specifically the Franseen and Fork-tip types, seem to provide superior specimen length and complete portal tract yield compared to 19G fine-needle aspiration (FNA) and smaller 22G FNB needles in EUS-guided liver biopsy. However, the low-quality evidence, due to bias and imprecision, requires further well-designed randomized controlled trials to confirm these results and inform clinical guidelines for needle selection in EUS-LB.

## Figures and Tables

**Table 1:** GRADE summary of findings reporting the comparative efficacy of different needles for EUS-guided liver biopsy.

	Total Specimen Length		Complete Portal Tracts	
	MD (95% CI)	Quality of Evidence	MD (95% CI)	Quality of Evidence
**All needles vs. 22G Franseen**				
19G FNA	−0.73 (−4.32 to 2.86)	Low	−12.40 (−29.27 to 4.47)	Low
19G Fork-tip	**2.76 (1.43 to 4.10)**	Low	9.92 (−2.49 to 22.32)	Low
19G Franseen	**3.19 (2.07 to 4.31)**	Low	9.10 (−0.98 to 19.18)	Low
22G Fork-tip	−2.02 (−5.95 to 1.91)	Low	−13.70 (−33.36 to 5.96)	Low
**vs. 22G Fork-tip**				
19G FNA	1.29 (−0.30 to 2.88)	Low	1.30 (−8.80 to 11.40)	Low
19G Fork-tip	**4.78 (0.95 to 8.62)**	Low	**23.62 (5.25 to 41.98)**	Low
19G Franseen	**5.21 (1.45 to 8.97)**	Low	**22.80 (5.92 to 39.68)**	Low
**vs. 19G Franseen**				
19G FNA	**−3.92 (−7.33 to −0.51)**	Low	**−21.50 (−35.02 to −7.98)**	Low
19G Fork-tip	−0.43 (−1.15 to 0.30)	Low	0.82 (−6.42 to 8.05)	Low
**vs. 19G Fork-tip**				
19G FNA	**−3.49 (−6.98 to −0.01)**	Low	**−22.32 (−37.65 to −6.98)**	Low

Abbreviations: CI, confidence interval; FNA, fine-needle aspiration; MD, mean difference. Mean differences reaching the significance threshold are reported in bold.

**Table 2:** Characteristics of included randomized controlled trials comparing different needles for EUS-guided liver biopsy.

Author, Year	Study Period; Country	Needles Used	N Patients	Approach	Aspiration Technique	Actuations	N Passes	Adequacy Rate	Total Specimen Length	N CPTs	Adverse Events
Ching-Companioni, 2019 [18]	2017; USA	FNA 19G vs. Franseen 19G	20 FNA 20 Franseen	Bilobar	Heparin wet suction	7–10 back-and-forth, fanning	2 (1 per lobe)	FNA: 14 (70%) FNB: 18 (90%) ^a^	FNA: mean 114 mm (SD 5.55) FNB: mean 153.2 mm (SD 5.24)	FNA: median 16.5 (range 6–38) FNB: median 38.0 (range 0–81)	FNA: 7 (35%) FNB 6 (30%) abdominal pain
Mok, 2019 [19]	2016; USA	FNA 19G vs. Fork-tip 22G	20 both needles	Bilobar	Heparin wet suction	7–10 back-and-forth, fanning	4 (1 per needle, 2 per lobe)	FNA: 35 (88%) Fork-tip: 27 (68%) ^b^	FNA: mean 61.0 (SD 30.4) Fork-tip: mean 48.1 (SD 32.2)	FNA: mean 7.4 (SD 5.9) Fork-tip: mean 6.1 (SD 7.2)	1 (2.5%) abdominal pain
Hashimoto, 2021 [20]	2018–2019; USA	Fork-tip 19G vs. Franseen 19G	22 both needles	Left (11) or right (11)	Wet suction	1 actuation	1 per needle	Fork-tip: 6 (27.3%) Franseen: 15 (68.2%) ^a^	Fork-tip: 34.6 mm (range 3–94) Franseen: mean 44.9 mm (range 10–78)	Fork-tip: mean 14.4 (range 2–33) Franseen: mean 9.5 (range 0–35)	2 (9.1%) abdominal pain
Aggarwal, 2021 [21]	2019–2020; USA	Fork-tip 19G vs. Franseen 19G	107 both needles	Left lobe	Heparin wet suction	3 actuations	1 per needle	Fork-tip: 79.5% Franseen: 97.2% ^c^	Fork-tip: mean 13.86 mm Franseen: mean 15.81 mm	Fork-tip: mean 7.07 (SD 3.77) Franseen: 9.59 (SD 4.70)	1 (0.9%) gastric hematoma
Diehl, 2024 [22]	2021–2022; USA	Franseen 19G vs. Franseen 22G	21 19G 21 22G	Bilobar	Heparin wet suction	3 actuations	2 (1 per each lobe)	Franseen 19G: 17 (81%) Franseen 22G: 2 (9.5%) ^a^	Franseen 19G: mean 5.49 (SD 1.6) Franseen 22G: mean 2.30 (SD 1.4)	Franseen 19G: mean 12.36 (SD 4.62) Franseen 22G: mean 3.26 (SD 4.62)	Franseen 19G: 3 (14%) Franseen 22G: 2 (10%) abdominal pain

Abbreviations: CPTs, complete portal tracts; FNA, fine-needle aspiration; FNB, fine-needle biopsy; SD, Standard Deviation. ^a^ Adequacy defined by specimens containing ≥11 CPTs; ^b^ Adequacy defined by specimens containing ≥5 CPTs; ^c^ Adequacy not defined by a specific number of CPTs.

**Table 3:** SUCRA ranking for total specimen length and number of complete portal tracts.

Total Specimen Length	SUCRA	Complete Portal Tract	SUCRA
19G Franseen	0.91	19G Fork-tip	0.85
19G Fork-tip	0.83	19G Franseen	0.82
22G Franseen	0.41	22G Franseen	0.53
19G FNA	0.24	19G FNA	0.17
22G Fork-tip	0.09	22G Fork-tip	0.10

## Data Availability

The original contributions presented in this study are included in the article/Supplementary Materials. Further inquiries can be directed to the corresponding author.

## Supplementary Materials

The following supporting information can be downloaded at: https://www.mdpi.com/article/10.3390/diagnostics16121857/s1, Table S1. Details of search strategy. Table S2. GRADE (Grading of Recommendations, Assessment, Development and Evaluation) categories of quality of evidence. Table S3. Diagnostic adequacy. Table S4. Ranking in terms of diagnostic adequacy. Figure S1A. Risk of bias graph: review authors’ judgements about each risk of bias item presented as percentages across all included studies. Figure S1B. Risk of bias summary: review authors’ judgements about each risk of bias item for each included study. PRISMA NMA Checklist of Items to Include When Reporting A Systematic Review Involving a Network Meta-analysis.

## Abbreviations

The following abbreviations are used in this manuscript:

AASLD	American Association for the Study of Liver Diseases
AE	Adverse Event
CPT	Complete Portal Tract
EUS	Endoscopic Ultrasound
FNA	Fine-Needle Aspiration
FNB	Fine-Needle Biopsy
GRADE	Grading of Recommendations Assessment, Development and Evaluation
LB	Liver Biopsy
MD	Mean Difference
PRISMA	Preferred Reporting Items for Systematic Reviews and Meta-Analyses
RCT	Randomized Controlled Trial
RR	Risk Ratio
SD	Standard Deviation
SUCRA	Surface Under the Cumulative Ranking Curve
USA	United States of America
